# Imaging Aluminum Particles in Solid-Propellant Flames Using 5 kHz LIF of Al Atoms

**DOI:** 10.3390/ma12152421

**Published:** 2019-07-29

**Authors:** Gautier Vilmart, Nelly Dorval, Robin Devillers, Yves Fabignon, Brigitte Attal-Trétout, Alexandre Bresson

**Affiliations:** 1 Département de Physique, Instrumentation, Environnement et Espace, ONERA, Université Paris-Saclay, F-91123 Palaiseau, France; 2 Département Multi-Physique pour l’Energétique, ONERA, Université Paris-Saclay, F-91123 Palaiseau, France

**Keywords:** planar laser-induced fluorescence, emission, aluminum atoms, aluminum combustion, aluminized solid propellant

## Abstract

Laser-induced fluorescence imaging of aluminum atoms (Al-PLIF) is used to analyze the spatio-temporal behavior of aluminized solid propellant combustion. Using alternating LIF and chemiluminescence emission images of the particles in the gaseous and liquid phase evolving close to and far above the dynamically varying propellant surface, sequences of images were recorded and analyzed. The good sensitivity achieved enabled us to track the dynamics of the flame in the vicinity of particles detected all along the flame extension and up to 1.5 MPa. Analysis of wide-field images enabled droplet velocity measurements due to the high LIF sampling rate (5 kHz). The observed typical plume structures were in good agreement with alumina-formation prediction and previous shadowgraphy visualization. High-resolution sequences of images showed gaseous distribution behavior around the molten particles. The Al vapor phase was thus found to extend between 3 and 6.5 radii around the particles. Particle detachment dynamics were captured just above the propellant surface.

## 1. Introduction

Solid propellants are commonly used for space and military applications due to their efficiency and low cost of application [1,2,3,4,5]. Good understanding of combustion behavior during the melting, evaporation, and ignition of solid propellant fuels is required to maximize fuel efficiency and to optimize the design of combustors. These fuels contain metals, binders, and other solid materials which are ejected as solid particles and droplets off the surface during the volatilization processes. Models can be found in the literature including the complex interplay between fuel volatilization, droplet formation, and evaporation and chemical modeling of the combustion process with heterogeneous kinetics [6,7,8]. The spatio-temporal evolution of droplet size, velocity, temperature, and vapor concentration during combustion are key parameters of interest which need to be measured quantitatively and non-intrusively by optical diagnostic techniques.

Optical diagnostics of propellant flames remain scarce. Techniques such as coherent anti-Stokes Raman scattering (CARS) using nanosecond lasers [9], or femto/picosecond lasers [10] have been applied to spatially-resolved measurement of gas temperature. Digital in-line holography gives droplet size and velocity measurement [11] at rather low pressure. Concerning laser-induced fluorescence (LIF), spatial distributions of AlO around a drop burning in a simplified atmosphere was reported by Bucher et al. twenty years ago [12]. Hedman et al. have recently performed high-speed (5 kHz) LIF mapping of OH in non-aluminized propellants burning up to 1 MPa [13]. To our knowledge, no LIF diagnostic of aluminum combustion in solid propellant flames has been reported. Al and AlO vapors were probed by absorption in a shock tube at 20 bar and 3000 K [14], and by emission spectroscopy in a flame at 1 bar and 3500 K [15]. Laser-induced breakdown spectroscopy (LIBS) was demonstrated very recently for detecting metal particles, including aluminum (Al), in metallized propellant flames [16]. Femtosecond-LIBS has also been compared to nanosecond-LIBS [17].

In our laboratory, metallic atoms such as iron and aluminum [18,19,20] have been probed. More particularly, laser-induced fluorescence imaging of aluminum atoms (Al-PLIF) at a high repetition rate was shown to be quite informative about particle evolution in the liquid and gaseous phases. Aluminum fluorescence signatures have particularly strong intensities [19,20]. The purpose of our measurement is to capture the dynamic evolution of the Al droplet combustion process close to the propellant surface and above, during the short combustion duration (~1 s) of ammonium perchlorate (AP) composite propellants loaded with aluminum particles. In our experiment, blackbody emission mainly originating from alumina (on top of flame chemiluminescence), and LIF of aluminum particles were recorded separately and compared. This challenging task was accomplished due to the KHz-repetition rate of short laser pulses (nanosecond), proper synchronization of image acquisition, and a good signal-to-noise ratio due to strong Al-atom signal strength which enhances the LIF sensitivity in solid-propellant flames (high-pressure, bright background-luminosity, multiphase flows).

In a first step, a simple visualization of certain dynamic combustion processes of solid propellant fuels (e.g., droplet velocity evolution and spatial distribution of gas-phase aluminum around droplet) is performed in our study. However, due to the large signal available in LIF, and to the comparison of several species behavior with computational fluid dynamic modeling of the flame, more quantitative physical information may be obtained in the future including the location of homogeneous and heterogeneous formation of alumina; the temperature gradient at the propellant surface, which is required to retrieve quantitative species concentration; or finally, the concentration of chlorinated species, for example, which are known to be present in large amounts in these flames [9].

In this paper, image recording was undertaken in the flame at total pressures of 1.2 and 1.5 MPa, with and without laser excitation (Section 3.1.1.). Both blackbody emission and LIF signals were evaluated through a short sequence of images. This revealed the presence of gaseous aluminum around the droplets, inside the molten particle halos, but also alumina blackbody emission which was spread all over. Interesting droplet behavior with a typical plume surrounding the molten particle was evidenced. Plume behavior was seen to be influenced by the dragging effect inside the hot flow, depending on particle velocity. Manual analysis, typically over one hundred successive images, was performed in order to follow several Al droplets, starting from the burning surface, and to calculate their velocity along the hot-gas region (Section 3.1.2.). In addition, automatic image processing was undertaken since a higher number of images could then be analysed (Section 3.1.3.). Evolution of droplet signals (LIF and emission) as a function of height above the burning surface is presented in Section 3.1.4. Finally, a high spatial resolution was used in the detection path in order to better observe and assign the physical processes taking place along the hot flow (Section 3.2).

## 2. Materials and Methods

### 2.1. Test Rig

Aluminized solid-propellant samples were burnt inside a pressurized chamber (to a defined level) by initially injecting nitrogen gas. The pressure was set to 1.2 and 1.5 MPa in the present experiment. Composite solid propellants are made of ammonium perchlorate (AP) particles gathered into a hydroxyl-terminated polybutadiene (HTPB) binder. Aluminum powder is added as a performance additive to improve and adjust effective impulse, with mass fractions up to 20%. A home-made research composite propellant (Al/AP/HTPB) investigated here. It contains 18 wt % aluminum particles, and the particle sizes were from 10 to 120 µm in diameter.

The test rig of solid propellant flames investigation was previously described in [18]. Small samples (height: 8 mm, width: 5 mm, thickness: 2 mm) are maintained in a holder which is placed at the center of the chamber. The long-duration pulse (0.225 s) of a CO_2_ laser (GL 2000, PRC Laser, Landing, NJ, USA) is used to ignite the samples. The CO_2_ laser beam irradiates the sample surface from the top of the chamber, at normal incidence through a ZnSe window. Propellant is heated by the laser (input power of 700 W) to reach ignition. The holder is kept fixed during the burning time so that the propellant regression is not compensated.

### 2.2. PLIF Setup

**Figure 1:**
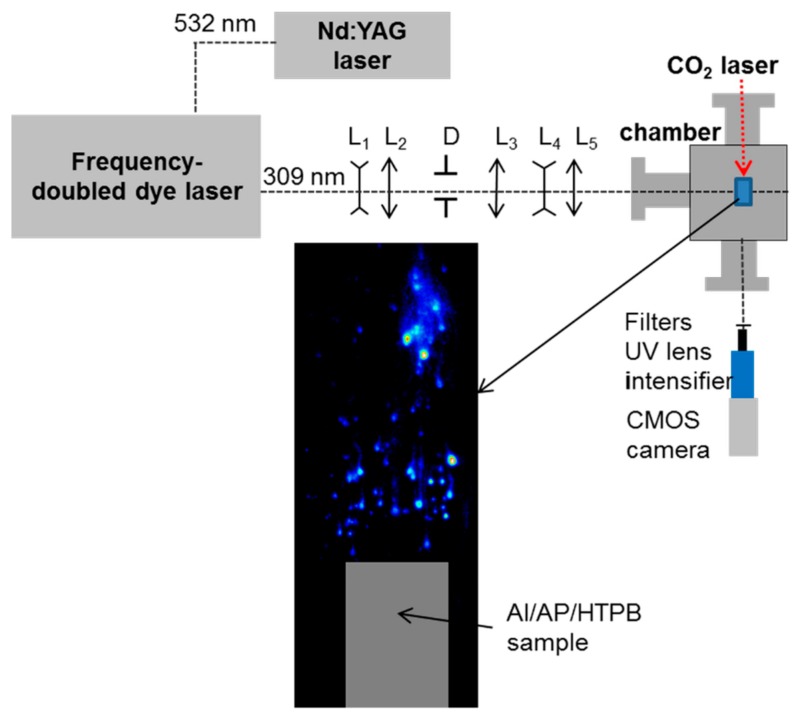
PLIF set-up and an example of laser-induced fluorescence imaging of aluminum atoms (Al-PLIF) image. The solid-propellant sample is displayed as a gray rectangle to suggest its location in the image. L: lenses for sheet formation. D: Diaphragm.

The laser and detection systems for the Al-PLIF setup are depicted in Figure 1. A tunable dye laser (Credo, Sirah Lasertechnik, Göttingen, Germany) is pumped by a Nd:YAG laser (INNOSLAB2011-E, EdgeWave, Aachen, Germany) operating at 532 nm. The high-repetition rate dye laser operates at 5 kHz and was described elsewhere [19]. The dye laser is frequency doubled in a BBO crystal and is tuned to the ^2^P^o^_3/2_−^2^D_5/2_ transition of Al (309.361 nm, vacuum wavelength). The wavelength is monitored by means of a wavelength meter (WS-600, HighFiness, Tübingen, Germany). The laser beam is expanded through a telescope (f = −50 mm and f = 200 mm) and the most intense and homogenous part of the expanded beam is spatially filtered by a pinhole. The beam is shaped into a laser sheet 17 mm in height and 0.1 mm in thickness through a combination of UV-fused silica lenses (f = 618 mm, f = −19 mm, f = 150 mm). Considering the laser beam area (17 × 0.10 mm^2^) and the pulse duration (5 ns), laser power density is estimated to be 225 kW·cm^−2^ at the probe volume. The laser sheet enters the chamber through a UV-fused silica window (clear aperture: 16 mm) and crosses the flame flow just above the propellant surface, as shown in Figure 1. The laser sheet crosses the flame from right to left in each of the images. Fluorescence light is amplified by a two-stage micro-channel plate image intensifier (HS-IRO, LaVision, Göttingen, Germany) and imaged by a CMOS camera (HSS6, LaVision, Göttingen, Germany). Spectral selection is achieved using two bandpass filters centered at 394 nm (FWHM = 10 nm) to recover indirect fluorescence lines at 394.512 nm (^2^P^o^_1/2_−^2^S_1/2_) and 396.264 nm (^2^P^o^_3/2_−^2^S_1/2_) [19,20]. A long-pass filter at 385 nm is also used in order to suppress laser light scattering from particles and smoke (including Al_2_O_3_ nanoparticles). A wide field of view (17 × 17 mm^2^) is obtained by using a Cerco f/4.1 94 mm lens and a set of spacers (total extent of 68 mm) to increase the magnification to about 1. The camera operates with 768 × 768 pixels, leading to 22 µm per pixel. High-resolution imaging (3 × 3 mm^2^ with 3.9 µm per pixel) is also performed with a magnification of about 5. For that purpose, a UV long distance microscope objective (QM-100, Questar, New Hope, PA, USA) is added to the intensifier/camera assembly. The depth of field is smaller in this later case (100 µm), which implies that droplets can easily get out of focus. The camera optic is set perpendicular to the direction of the laser beam (Figure 1). Since the surface regression is not compensated during the combustion run, the field of view of the camera is set in order to cover the height of the propellant sample (5 mm) and an upper part above the surface (11 mm). This way, the burning surface is monitored during the whole combustion run. 

Images were recorded at 10 kHz repetition rate (0.1 ms frame-to-frame interval) which is twice the laser frequency. This way, alternating images with the laser switched on/off were recorded during all combustion runs. One frame in two showed either the emission background from the hot gases and particles (without laser) or the fluorescence signal itself (with laser) on top of the emission background. Elastic light scattering could be identified and isolated from fluorescence signal by recording images when the laser wavelength was tuned off resonance in a dedicated combustion run; the later images were compared to all other images recorded without laser. In this way, elastic scattering contribution was found to be negligible (Section 3.1.1.). 

The intensifier gate width was set to the minimum value of 100 ns. An external signal delivered by the ignition laser (CO_2_) was used to trigger the intensifier gate so as to synchronize and tag the probed images with regard to the initial time (t_0_) of the combustion sequence. 

## 3. Results and Discussion

### 3.1. Wide Field of View

#### 3.1.1. LIF and Emission Image Analysis

Spectral investigation was performed in the UV range in order to reduce interference with the blackbody radiation. The fluorescence signal was detected far away from the excitation wavelength to filter elastic light scattering. Prior to Al-PLIF measurements in solid-propellant flames, spectroscopic studies have been performed under steady-state conditions inside two dedicated evaporation chambers, namely a resistive heater evaporation chamber and a laser vaporization chamber [19,20]. Both systems were used in order to validate our excitation/detection scheme (309/(394–396) nm) and to quantify the pressure and saturation effects on signal behavior. A theoretical model including quenching and saturation effects has been elaborated in [20]. The model was validated through a number of experiments, among them fitting the spectral excitation profiles with computed profiles and measuring the fluorescence decay time to retrieve collisional parameters, in a dedicated experiment in the resistive heater evaporation chamber. A fluorescence quantum yield of 2% at 0.1 MPa (N_2_) and 2500 K was measured, which was extrapolated to 0.15% at 1.5 MPa.

**Figure 2:**
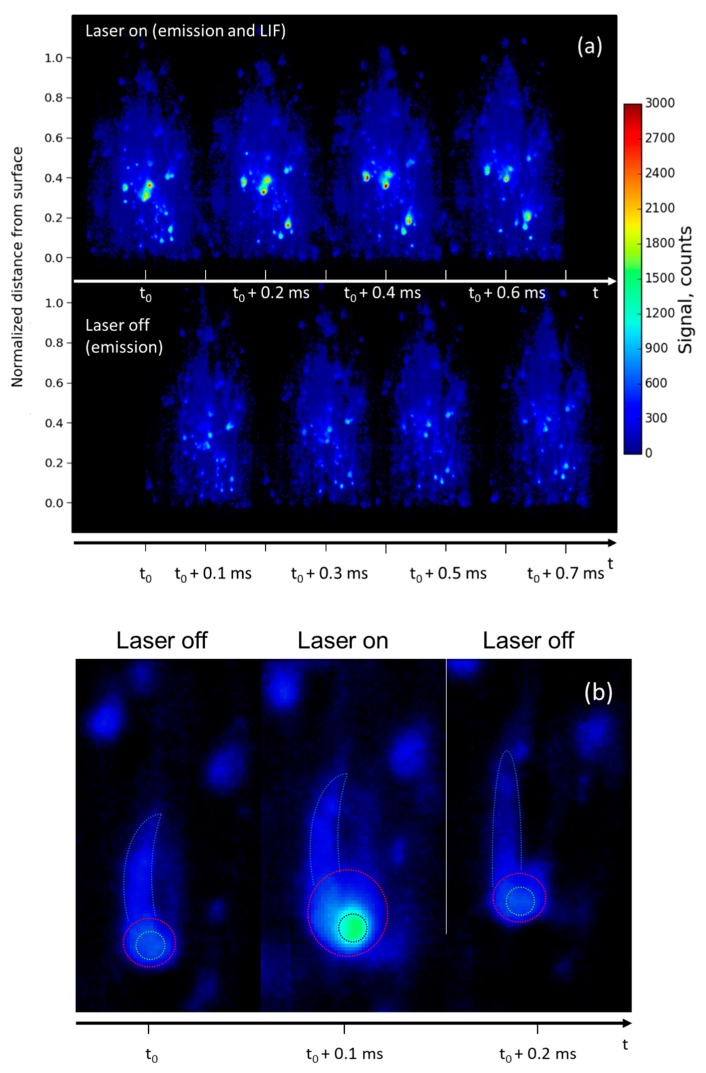
(**a**) Comparison between two sequences of four instantaneous images of laser-induced fluorescence (LIF) signal (with laser on, top sequence) and emission signal (without laser, bottom sequence). The distance from surface is normalized to the total flame height. The time delay between successive images is 0.1 ms. The time delay between LIF images is 0.2 ms. The sequence duration is 0.8 ms. Images are recorded during the combustion of a burning Al/AP/HTPB propellant sample at 1.2 MPa. The dynamic of the signal is 3000 counts; (**b**) time sequence of three successive expanded images of a droplet extracted from Figure 2a at h = 3 mm. The color range is white for the particle core, red for the vapor extension, and blue for the plume tail.

We recall here that the image acquisition frequency was twice the laser repetition rate. Two sequences of four images are presented in Figure 2a as a function of time. The top sequence shows the images recorded with the laser on and the bottom sequence shows background emission (without laser). Expanded views of a luminous particle observed in both emission and LIF images are shown in Figure 2b. Let us recall that the laser sheet crossed the flame from right to left in each image. Let us also note that in all the spatio-temporal images (distance versus time), distance to the surface has been normalized to the total flame height.

Allocation of the evaporating Al droplet was made possible by comparing the alternating emission and LIF image sequence. The LIF signal was observed in the top sequence (around 3000 counts) originating from some droplets in the flame (bright spots) emanating from the burning surface, whereas the emission level was lower (750 counts). The resulting signal-to-noise ratio (SNR) was four. As a matter of fact, the LIF signal (laser tuned on resonance with the Al transition) was unambiguously attributed to a liquid Al droplet surrounded by a cloud of gaseous Al atoms evaporating at high temperature during and after ignition. The strong intensity of the LIF signal resulted from the high density of atoms in liquid particles burning at high temperature. 

In such a multiphase flow, discrimination between solid, liquid, and vapor phases is not straightforward. However, the LIF signal originating from the liquid phase will be far more intense than the gas-phase signal according to the relative atom number density (superimposed signals). Moreover, the LIF signal is a good marker for phase transformation of the Al particle from its solid phase to its liquid phase (melting point of 930 K) and to evaporating Al (boiling point of 2745 K), which is burning. The flame is located in the droplet area since evaporating Al atoms react with the ambient gases [21,22] to form new species including alumina (Al_2_O_3_). Our imaging technique enables Al transformation inside the combustion to be monitored. 

On the other hand, emission radiation mainly results from the thermal emission (blackbody radiation) of the hot particle surfaces, from the gas-phase flame, and from oxide smoke (Al_2_O_3_) forming clouds dispersed in the flame. Al_2_O_3_ is assumed to be formed and to be molten at first (melting point at 2300 K). According to a new theoretical combustion prediction [21,22] a homogeneous alumina nucleation process is taking place inside the hot flow around the aluminum droplets (which can be very small) by oxidation of the Al gas. Chemical phase transformation ends when temperature decreases so that species condensate. Although it was not done here, emission radiation may be studied in more detail spectroscopically, to reveal the chemical nature of all irradiating species [20]. 

The images in Figure 2a exhibit a weak and inhomogeneous background (light blue) spread all over the flame (300–400 counts). The background is stronger in the vicinity of the fluorescent particles and extends across the whole flame. It results from radiation of hot gases and also originates from the hot and fine alumina (Al_2_O_3_) smoke. Al_2_O_3_ nanoparticles are spread in the flow as soon as they are formed on the Al droplet surface by the oxidation reaction, thus contributing to the luminous flame background.

Allocation of the different phases (solid nanoparticles, liquid droplet, gaseous Al) was made possible by comparing the alternating emission and LIF images sequences displayed in Figure 2b. The droplet core is allocated in emission at t_0_. Then, it strongly fluoresces in the middle image (SNR of four) due to the presence of a high density of aluminum, in its liquid and gaseous phases. The central zone is circled in white and does not change in size through the three displayed images. Around the mostly liquid droplet core (visible on the emission image), the larger extension of the LIF signal unambiguously allocates gaseous Al atoms, which extend to fill the red circle with some additional clouds upward and downward also exhibiting fluorescence, although barely visible. By comparing the mean LIF-signal level measured in this area (250 counts) to the one obtained in controlled experimental conditions with a known density of atoms, and by using the model described in [19] to account for the differences in pressure, temperature, and laser intensity, the density of gaseous atoms was estimated to be about 10^12^ cm^−3^.

The smoke emission (circled in blue) above the droplet narrows upwards to form a plume. Although not exactly identical because of the time delay, it has very similar behavior in the three images, showing that nearly no gaseous aluminum is present in this region, and providing a sketch of its evolution. Each image from Figure 2b shows that smoke emission and fluorescence are dragging upward, since both Al vapor and fine Al_2_O_3_ nanoparticles flow inside the flame gases [21,22]. Nevertheless, the velocity is moderate (Section 3.1.2.). Finally, due to the very short duration of Al atom fluorescence (<10 ns) that freezes the physicochemical changes of the lightning particle and the recording of alternate images, it is possible to identify gaseous Al-atom location with a high spatial and temporal resolution. 

**Figure 3:**
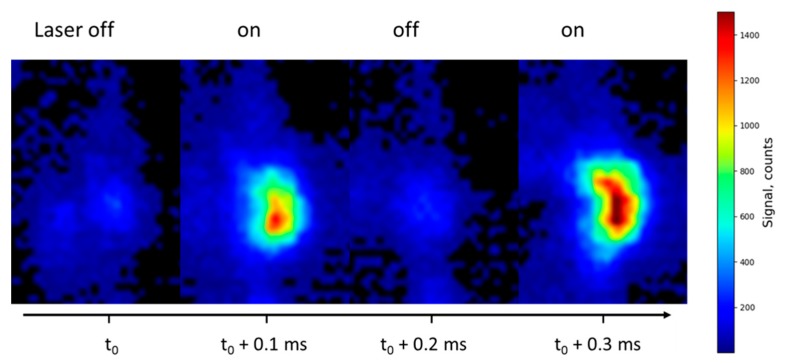
Alternation of instantaneous images, with laser switched off/on, recorded during the combustion of an Al/AP/HTPB solid-propellant sample at 1.5 MPa. One frame over two shows the contribution of the emission background. Sequence duration is 400 µs. The bean-shaped LIF island is isolated from the right-hand side of the flame, probably resulting from agglomeration. The dynamic of the signal is 0–1500 counts.

Figure 3 presents a sequence of four images of the same burning propellant at a higher pressure of 1.5 MPa with and without laser. As the pressure increases, the LIF/emission signal ratio becomes smaller. The bean-shaped LIF particle observed (on the right-hand side of the flame) is seen to produce a huge LIF signal, likely due to its higher temperature and larger size, probably as a result of an agglomeration process. 

**Figure 4:**
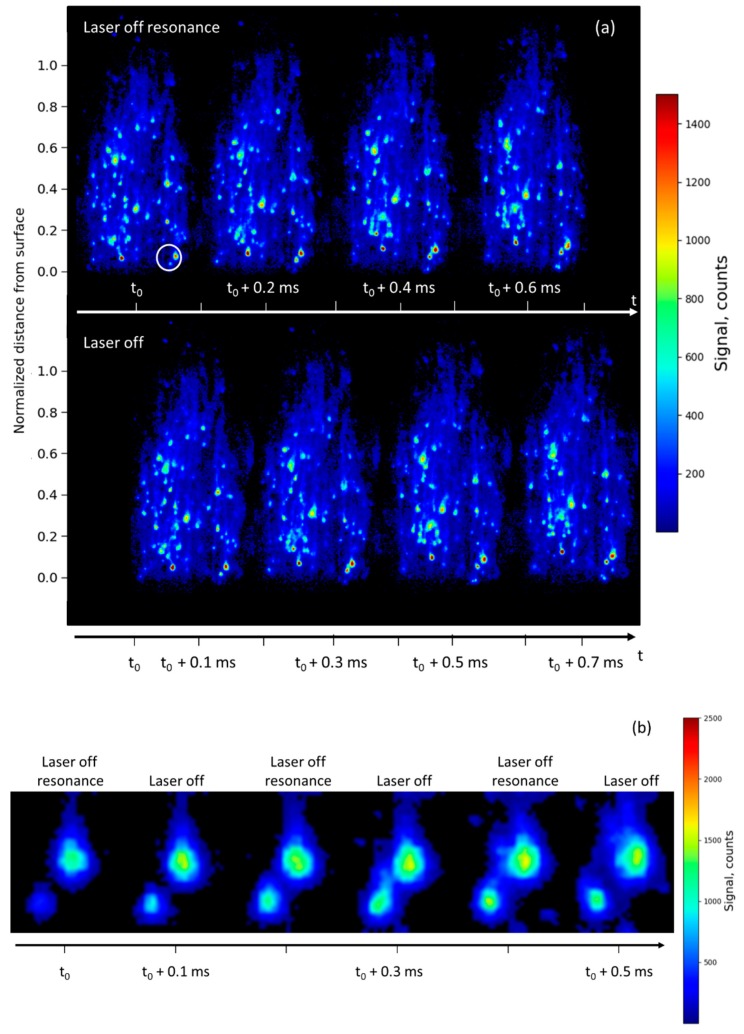
(**a**) Comparison between two sequences of four instantaneous images of laser scattering signal (with laser off resonance, top sequence) and emission signal (without laser, bottom sequence). The distance from surface is normalized to the total flame height. The time delay between successive images is 0.1 ms. The sequence duration is 0.8 ms. Images are recorded in an Al/AP/HTPB solid-propellant sample burning at 1.5 MPa; (**b**) expanded views of a couple of droplets extracted from Figure 4a (white circle); sequence duration of the film is 0.5 ms in this case. The dynamic of the signal is 0–1500 counts.

For the majority of droplets, the contrast becomes lower as pressure increases. A background emission (light blue) is still observed in Figure 4, all over the flame. It is as intense as in Figure 2 and the emission remains restricted to the alumina particles region, all shaped as plumes.

In Figure 4, the laser is detuned far away from Al absorption line center (by 500 pm, laser off resonance). In Figure 4a, four images are recorded with laser off resonance (upper sequence) and four emission images are recorded without laser (bottom sequence). Similar signal levels are obtained in both cases confirming that elastic light scattering from particles is small. Hence, only light emission from gas-phase and condensed/molten particles (including nanoparticles) interfered with the LIF signal from Al atoms.

A close-up sequence is presented in Figure 4b, by extracting expanded views of a droplet (white circle in Figure 4a). It does not matter that the laser is on and off because spontaneous emission is dominant in all images, as already mentioned. However, it is interesting to observe that the signal changes slowly enough that the sampling rate is sufficient to provide a good insight into the phase transformation process, even though the sampling rate is not high enough to follow the chemical reactions. The latter have reaction rates typically in the range 10^−6^–10^−9^ s, and even shorter, whereas the frame-to-frame time interval of 10^−4^ s is available with our laser system [7]. In Figure 4b, the signal evolution of both particles is mainly driven by temperature changes and is seen to increase versus time with smoke production during the combustion process (increasing halo).

#### 3.1.2. Droplet Trajectory and Velocity Measurement

Such a high-speed Al-PLIF technique is a powerful tool since analysis of the images gives access to important information such as:

- Trajectories of the molten aluminum droplets;
- Velocities as a function of distance from the propellant surface;
- Lifetimes of the Al droplets evolving in the flame from the moment of fluorescence signal appearance (probably ignition) to its extinction, providing a way to measure their burning properties.

**Figure 5:**
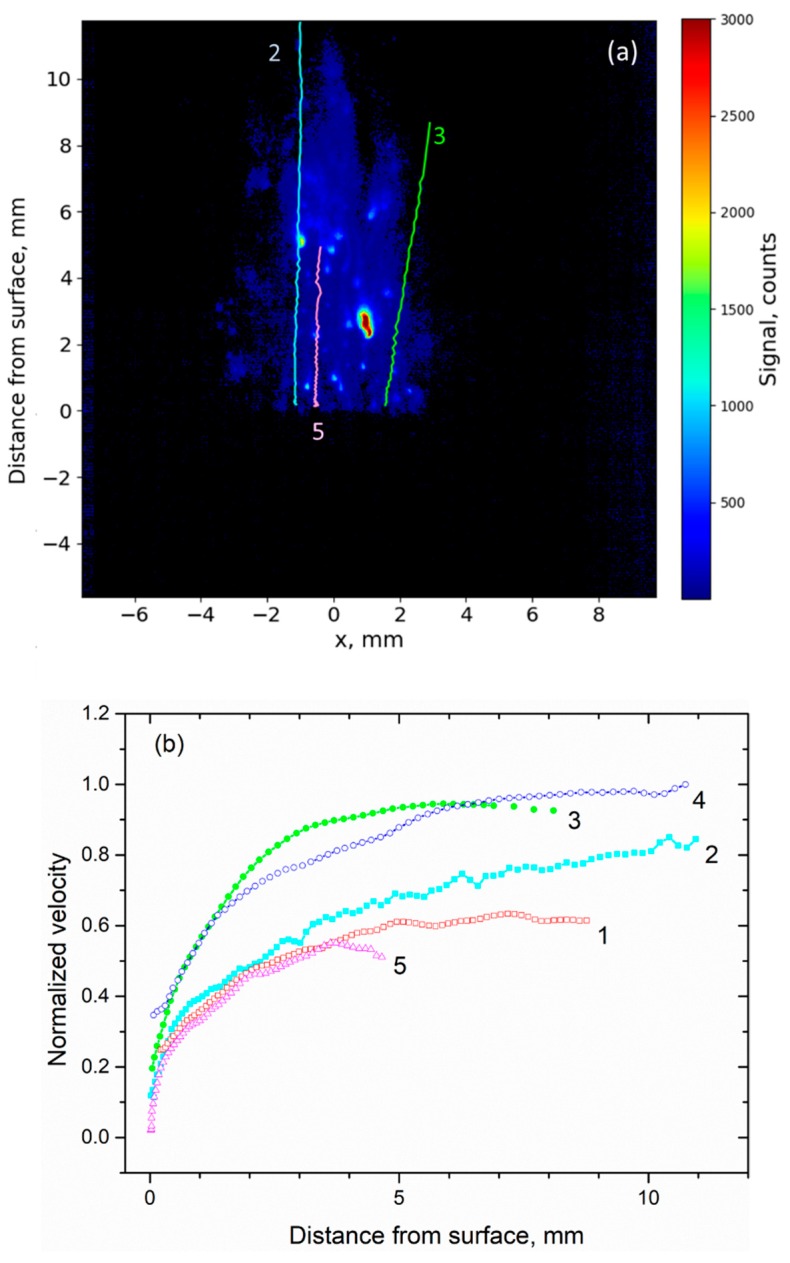
Analysis of droplet behavior following the flow: (**a**) Trajectory of Particles 2 (light blue), 3 (green), and 5 (pink); (**b**) Normalized velocity versus distance above the burning propellant surface (Al/AP/HTPB) at 1.2 MPa. Velocity is measured for five different particles. Smaller particles have steeper velocity increase in the first mm.

The potential of our technique for measuring droplet trajectory is illustrated in Figure 5. The location of a fluorescent droplet is followed manually from one image to another. The resulting droplet trajectories for three particles are shown in Figure 5a. The measurement repetition rate is sufficiently fast to allocate two successive positions of the droplet (according to its velocity). Between two consecutive images, a droplet would move by only a few pixels. Given the size of the image (17 mm = 768 pixels), it is thus possible to follow a droplet over about 100 images (i.e., over 10 ms). Temperature rises just above the propellant, and particles are molten all across the flame. The trajectory of Particle 2 is remarkably straight and vertical (light blue). The droplet is still visible up to 11 mm above the propellant surface due to its size and brightness. The solid-propellant burning surface location is measured on the image (followed on a camera).

The vertical velocity of a particle is retrieved by measuring its positions in the images and differentiating these. To illustrate the previous procedure, the resulting velocities of five particles are shown in Figure 5b. All droplets except droplet 5 are located on the lateral border of the flame. First, particles are extracted from the surface of the propellant either with a velocity close to zero (particles 2 and 5) or already with some initial speed (particles 1, 3, 4). Either way, they then experience acceleration during the first 2 millimeters following release (< 4 mm). The velocity profiles are strongly dependent on their size due to the drag effect. Only smaller particles are able to follow the flow whereas larger ones are slowly dragged. The two particles exhibiting a steep acceleration over the first mm and the highest velocity are expected to be the smallest. Velocities reach a plateau over the last 7 mm. This asymptotic value depends on the droplet size, assuming that flame temperature and gas velocity are unchanged.

Al-droplet velocity measurements were also performed using the shadowgraphy technique for—the same solid propellant flame burning at 1.2 MPa. Shadowgraphy images are limited to a field of view of only 3 × 3 mm^2^ and recorded with a lower sampling rate (from 3 to 6 kHz). The larger field of view and higher sampling rate of the LIF technique improve the trajectory analysis and enlarge the measurement domain, while being chemically selective. Good agreement was previously found between the two measurement techniques [23]. 

Droplet size was measured with shadowgraphy imaging with, as previously noted, there being a strong correlation between velocity profiles and droplet size. In the LIF image, the signal originates both from liquid and vapor, the latter enlarging the particle spatial extension, so that the method is not well adapted to droplet sizing. Nevertheless, comparing the velocity profiles drawn from emission/LIF (Figure 5b) and shadowgraphy, it is possible to evaluate the size for previous droplets in the range of 80–170 µm as previously published [23]. The diameters estimated from emission images were in the 150–260 µm range for particles 1 to 5; thus the particle size is definitely attributable to its hot and luminous center. One can notice that emission images overestimate the size of the droplets (1.8 r_0_), as does LIF to an even greater extent, therefore confirming that the gaseous phase has a noticeable contribution to both images (see Section 3.2).

#### 3.1.3. Statistical Analysis for Droplet Velocity

**Figure 6:**
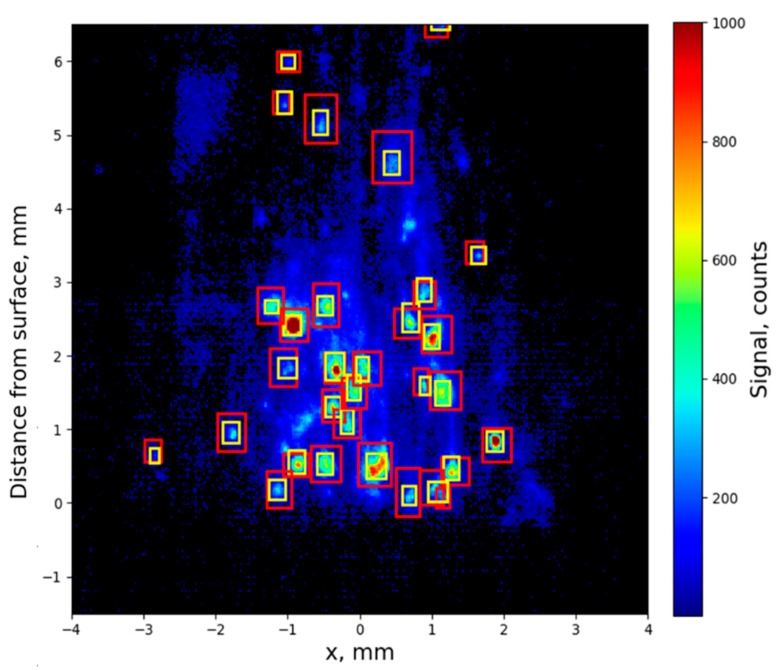
Zoom on the central part of the flame. The yellow boxes give the position of the manual spotting of the droplets and the red boxes display the result of the maximally stable extremal regions (MSER) algorithm treatment.

In order to increase the validity of the analysis, a large number of trajectories had to be reconstructed to statistically describe the droplet velocity across the full flame height, and throughout the full combustion duration. Because of the large amount of data to process, analysis involved using algorithms to automatically detect droplets and reconstruct their trajectories, (approximatively 700,000 droplets for a two-second experiment). Preliminary results of the droplet detection algorithm applied to the above propellant images are presented in this section (for a combustion test at 1.2 MPa). Because particles were detected over a long period of time, regression of the surface cannot be disregarded. Therefore, prior to using the droplet detection algorithm, the position of the solid-propellant burning surface was evaluated over time and the image was vertically translated so that the surface stayed at a fixed level on the vertical scale (at pixel 700 here). This provides a common spatial reference for all images. The algorithm used to detect particles is the maximally stable extremal regions algorithm, namely MSER [24]. This was previously used for droplet detection of shadowgraphy images [23]. It consists of binarizing the images with different signal-level thresholds and detecting the spatial regions whose shape remains stable. The algorithm application is illustrated in Figure 6. It was used in the current study to retrieve the position of the particles.

Several parameters affect the sensitivity of the MSER algorithm. Parameters were adjusted by comparing MSER prediction with manual spotting of the droplets. The goal was to detect the most hand-picked droplets (i.e., retrieve as many “real droplets” as possible) without generating a high number of false negatives. The best performance level was achieved using different parameters for the LIF and emission images. This probably resulted from the fact that the LIF images contain droplets with a very strong signal, leading to an increase of the image contrast and a flattening of the less intense droplets. Overall, 85% of the hand-selected particles were detected by MSER, with a 16% false-positive rate. This is a very good level of detection performance. The false-positive rate was much better than that usually obtained for shadowgraphy-image analysis (in the range 40–55% in [23]).

**Figure 7:**
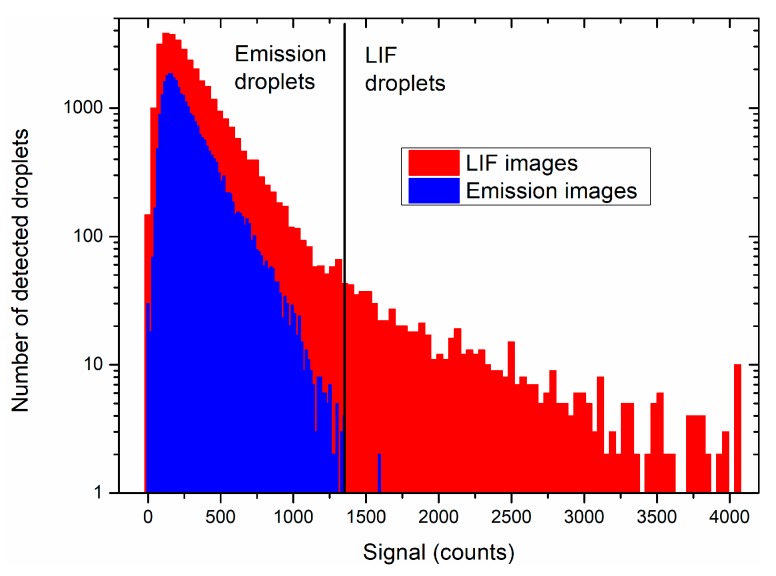
Signal distribution of the droplets detected by the MSER algorithm in the LIF and emission images.

Applying the algorithm to the whole combustion run (2700 images) yielded a total of 64,000 detected objects, one droplet being detected several times on successive images. The distribution of droplets as a function of signal level is shown in Figure 7 for the LIF and emission images. More droplets are found in the LIF images than in the emission images (34,000 vs. 30,000), mainly due to the algorithm being more sensitive in the first case, as previously discussed.

The droplet emission signal did not exceed 1400 counts (Figure 7; blue lines) in the emission images. Therefore, droplets exhibiting a signal higher than 1400 counts in the LIF images were only the fluorescing ones. This threshold yielded 690 fluorescent droplets, compared to 63,310 droplets obtained in emission (summing the emission droplets in the LIF and emission images). Thus, it was an easy initial way to separate the LIF droplets from the emission droplets inside the LIF images, though by no means exhaustive. Indeed, many LIF droplets had a signal below 1400 counts. A better way of distinguishing the LIF and emission droplets would be to reconstruct the droplet trajectories and check for the increase in signal of the droplets in the LIF images. This is the method that will be preferentially used in the future.

**Figure 8:**
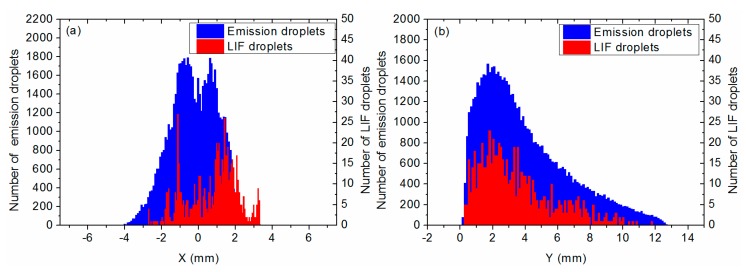
Horizontal (**a**) and vertical (**b**) distributions of the number of LIF and emission droplets for the Al/AP/HTPB sample burning at 1.2 MPa.

Since the MSER algorithm gives the positions of the detected droplets, it was possible to provide the horizontal and vertical distributions of the number of emission and LIF droplets, as shown in Figure 8.

The horizontal distribution extends from 2 to 4 mm for a total of 6 mm in Figure 8a. The initial width of the propellant sample is 5 mm. The distribution of emission droplets exhibits a hole in the flame center with a loss of about 30% of detected droplets. This might be due to the higher density of smoke in that region, which may attenuate the droplet radiation signal. Interestingly, the distribution peaks are different for the emission and LIF droplets: the emission distribution peaks at 0 mm whereas LIF distribution peaks at 1.5 mm. This was attributed to laser sheet absorption by the smoke as it propagated through the flame. 

The vertical distribution extends from 0 to 12.5 mm in Figure 8b. The number of droplets increases from 0 to the peak value reached at 1.9 mm, and exponentially decreases to 12.5 mm. The increase from the surface to h = 2 mm lasts 2–4 ms. The very first droplets leaving the surface are seen over a very short distance, 200–500 µm. The LIF and emission distributions reach maxima at the same distance from the surface. The location of the maximum probably corresponds to a region in which temperature is higher, on average, since dilution by surrounding N_2_ in the combustion chamber reduces hot-gas temperature further away from the solid-propellant burning surface. The signal is persistent until the aluminum is totally consumed, the lack of fuel lowering the reaction rate [21,22]; thus, very few LIF droplets can be seen above 10 mm from the burning surface (reached after about 10 ms spent in the hot-gas flow). This should indicate that the combustion of the droplets is over at this height. This will be verified with a more robust and accurate image analysis procedure including a better distinction between LIF and emission droplets, which will enable more LIF droplets to be detected. Experimentally, we also plan to increase the intensity of the laser and to shorten the duration of the intensifier gate from 100 to 40 ns, in order to increase the LIF intensity and better eliminate emission, thus simplifying the image analysis. 

#### 3.1.4. Vertical Evolution of the LIF and Emission Signals

**Figure 9:**
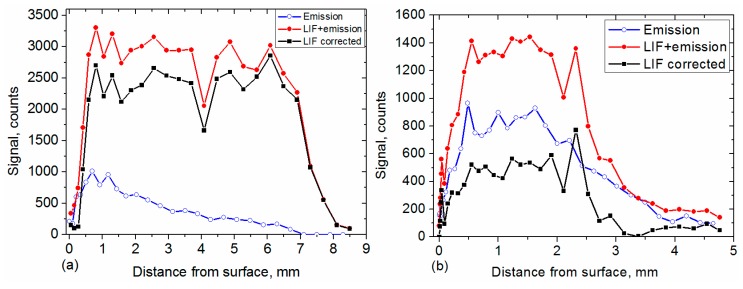
Comparison between emission (**○**) and LIF + emission (●) signals originating from (**a**) Particle 3 and (**b**) Particle 5 of Figure 5, versus distance above the burning propellant sample (Al/AP/HTPB) at 1.2 MPa. The LIF signal (■) is obtained after subtracting emission background and is corrected for the spatial non-uniformity of the laser sheet.

The evolution of the signal with and without laser is shown as a function of distance above the burning surface in Figure 9. Particles 3 and 5 were chosen for this study since they exhibited different velocity profiles in Figure 5. However, analysis of their behavior is representative of many other particles among the large number observed and treated in our images, due to the above treatment. 

Signal amplitude was averaged over an area of 3 × 3 pixels located in the brightest zone of the droplet. The emission part of the (LIF + emission) signal (●) can be removed by interpolating the emission intensity (**○,** recorded at a delay of 100 µs) and subtracting it from the LIF + emission signal, thus providing the pure LIF signal. Background-subtracted Al-PLIF signal was divided by the laser mean profile recorded from the acetone-fluorescence image to correct the LIF signal for the spatial inhomogeneity over the laser-sheet height. Signal was thus corrected for the spatial distribution of the laser energy (■). Comparisons between the vertical profiles of emission and LIF signals shown in Figure 9, provide clear evidence of frame-to-frame fluctuations of the LIF signal, whereas the emission signal is more stable for Particle 3 but fluctuates for Particle 5. Fluctuation of the LIF signal partly results from shot-to-shot fluctuations in laser power (in the range of ± 15%). In addition, larger fluctuations may originate from the location of the droplet inside the laser sheet thickness on top of the physical fluctuation resulting from combustion itself. The latter is indicated by emission which simply follows temperature. The laser sheet is currently 100 µm thick. As expected, the laser sheet location is subject to beam steering resulting from the gradient of the refractive index induced by temperature gradients. The unsteady nature of the flame may induce an intermittent pulsation of the laser sheet. Therefore, the droplet may be just inside the laser sheet at one point and slightly out of it at the following point. Signal alternation between “in” and “slightly out” may appear on the vertical profile since the map flips with the flame. Therefore, the LIF signal may also change accordingly, as sometimes seen in Figure 9. We plan to use a thicker laser sheet in future experiments to overcome this artefact. 

Particles are first ejected in the hot-gas flow and then start burning by emitting fluorescent light from both the liquid and surrounding gas phase, but with strong contrast. The vertical profile shows that LIF signal is already visible at a height of 200 µm and then increases significantly, as observed in Section 3.2. The signal level is driven by ignition, and thus temperature, across the flame. In the case of the fast (and small) Particle 3 (green, Figure 5a), the LIF signal is strong and constant, indicating a high Al concentration which is persistent because the particle is leaving the flame at the side, thus maintaining a constant liquid concentration. For the slower Particle 5 which is located near the flame center, the LIF signal is smaller with roughly the same level of emission (800 counts) as Particle 3, indicating less liquid (and gaseous) Al percentage inside the hot and burning particle (pink, Figure 5a) that already extinguishes at 4 mm in height.

In Figure 9, emission and LIF signals reach a maximum value between 0.5 and 1 mm above the burning surface. This is consistent with the distribution of fluorescent droplets and emitting droplets obtained in Figure 8b. 

### 3.2. High Resolution Imaging

**Figure 10:**
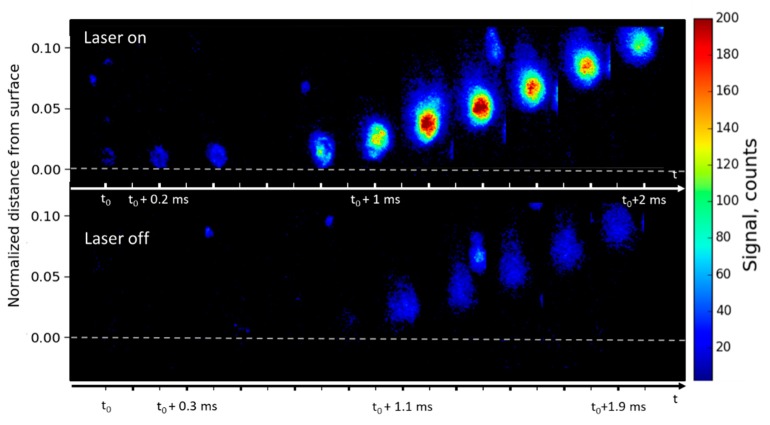
Sequence of eleven LIF images (top) and of ten emission images (bottom) just above the propellant surface. The distance from surface is normalized to the total flame height. The time delay between successive images is 0.1 ms. Images are recorded during the combustion of a burning Al/AP/HTPB propellant sample at 1.0 MPa. The propellant surface is indicated by a dashed line. The dynamic of the signal is 0–200 counts.

With a higher spatial resolution, it becomes possible to analyze the evolution of a single droplet, starting from a distance very close to the propellant surface. Analysis of the gas-phase spatial extent is obtained more accurately, and pertinent information can be highlighted. In particular, the location of Al vapor during the inhomogeneous particle transformation was evidenced. A sequence of eleven LIF images (i.e., with laser) is presented in Figure 10, showing a droplet leaving the surface of the Al/AP/HTPB sample burning at 1.0 MPa. In the images, the droplet can be followed during 2 ms (corresponding to eleven LIF images) and over 1.1 mm above the propellant surface. The emission images (without laser) are also shown. The Al droplet emits fluorescence but without emission signal as soon as it appears on the burning surface, where it remains for 600 µs before being detached from the surface. The surface position is located just under the stagnant particle and is indicated in Figure 10 as a dashed line. It is commonly assumed that the particles are preheated on the burning propellant surface yielding to the melting of aluminum on the surface—Al melts at 930 K, AP melts around 830 K, the binder reaches 1300 K, and the burning propellant surface is in the range of 800 to 1200 K for common solid-propellant compositions [25]. It is thus interesting to observe the aluminum particles very early in this thin preheated surface layer. In Figure 10, the Al particle can be seen emerging at a site on the surface before being released in the gas flow. Due to these high-resolution images, their residence time could be evaluated before they detached from the surface and was found to be less than 1 ms. Stagnation of aluminum at the surface is known to be a key issue for the formation of agglomerates [26]. The fast heating of the particle and its residence time duration on the surface can be related to the rise time of the LIF signal (as shown just after). The particle leaves the surface and is carried away by the upward gas flow. The process starts with a velocity equal to 0 and reaches its maximum value before the particles leave the field of view. The velocity profile drawn here is consistent with those previously shown in Figure 5. 

**Figure 11:**
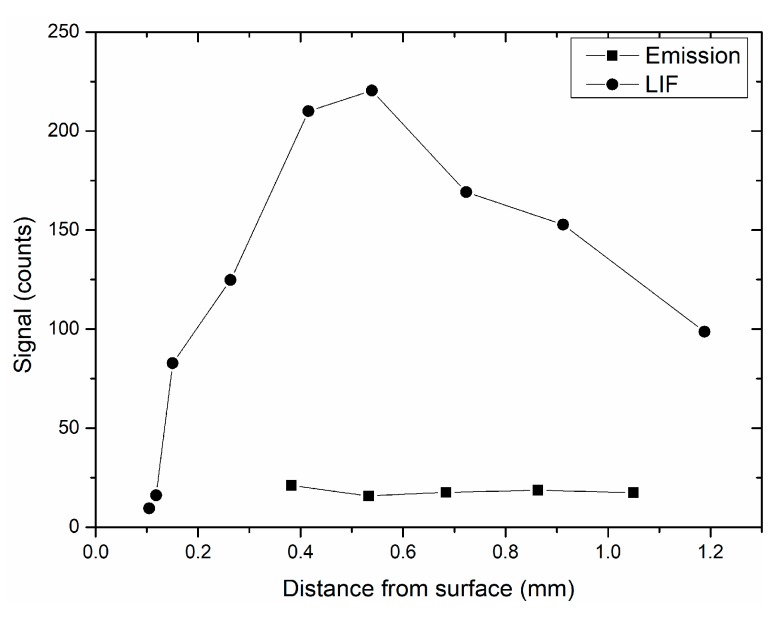
Vertical profiles of LIF and emission signals originating from the droplet observed in Figure 10. Signal averaged over a 3 × 3 pixel box on both LIF and emission images along the height.

The amplitudes of LIF and emission signals from Figure 10 are plotted as a function of distance above the surface in Figure 11. A steep rise of fluorescence signal level is observed up to 0.4 mm above the surface, consistent with the observation of Figure 9 (Particle 5) although height is more accurately known in Figure 11. Fluorescence decreases for height above h = 0.5 mm. No emission signal is observed over the first 0.4 mm. Emission signal appears at the time corresponding to the droplet’s detachment from the surface (after 1 ms). This typical behavior of the droplet “on the surface” was observed in several sets of images and is clearly reproducible. It remains constant until the droplet leaves the field of view. Since emission light is closely related to temperature, the evolution of the signal seems to indicate that particles are quickly heated near the surface due to a steep temperature gradient. In contrast to the weakness of emission signal, Al droplet fluorescence is substantially larger (SNR of 15 at 0.5 mm and 6.5 at 1 mm), making the droplet clearly visible in the images. Very near to the surface, emission is negligible. This is due to the depth of field of the objective, which is much smaller here (100 µm vs. 5 mm in the previous case of wide field of view). This drastically reduces the integration depth of emission signal, whereas the laser thickness is unchanged providing a better LIF/emission contrast than the previous wide field of view. The rise of LIF signal level is a reproducible scenario, indicating that temperature becomes high enough to produce molten Al particles, since particle enters into the flame zone and in a region where combustion starts (h = 0.5 mm). The amplitude of LIF signal sometimes decreases above 0.5 mm, although temperature is assumed to be constant in this zone according to the constant emission level. This indicates that another effect, such as misalignment, is taking place here. The moving droplet leaves the laser sheet, which is vertical and only 100 µm in thickness, since it is ejected in a tilted manner. Therefore, steering effects may explain the drop in the LIF signal between 0.4 and 1 mm.

**Figure 12:**
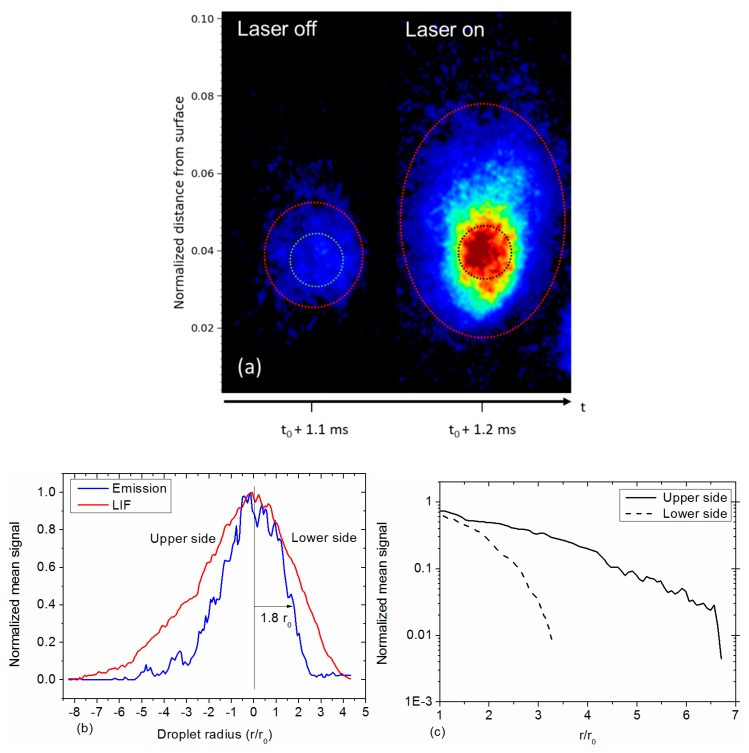
(**a**) Expanded views of the brightest droplet imaged in Figure 10 at h = 0.4 mm. The color range is white for the particle core and red for the vapor extension. The distance from surface is normalized to the total flame height; (**b**) Vertical profiles of LIF and emission signals as extracted from (a); (**c**) Vertical spatial extension of LIF and emission signals in log scale.

Expanded views of the brightest droplet at h = 0.4 mm are shown in Figure 12a. The LIF image is compared to the emission image extracted from Figure 10. The emission signal is ten times weaker than the LIF signal. A comparison between the two images readily shows the spatial extent of the gaseous aluminum in the region between the black and red dotted lines in the LIF image. Compared to Figure 2b, the reduced emission signal and the larger spatial resolution enable improved allocation of the gaseous distribution. Alumina smoke is not readily observable in these extended views. This may be due to the fact that the particle is in the process of ignition, and thus does not yet generate smoke in large amounts. It is also possible that the smoke is emitting too weakly to be detected in this improved SNR observation arrangement. In any case, the Al gaseous distribution becomes clearly visible, and additional improvements to the experimental system are in progress to better visualize the first step of the combustion process that takes place over the first mm above the burning surface and beyond. The SNR is definitely an important parameter to provide a better insight into the ignition moment when LIF is strongly emitted by particles, whatever their size.

Normalized vertical profiles of LIF and emission signals across the droplet are shown in Figure 12b. The droplet is limited to the core region (r_0_) in the LIF image corresponding to the strongest intensity and according to the size calibration done previously (see Section 3.1.2). The emission profile extends up to 1.8 r_0_ (at 1/e). It should be noted that the challenging alignment of the high-resolution setup makes the measurement delicate. Here again, improvements are planned for the alignment procedure using specific targets on the propellant surface. The vertical profile exhibits a long extension in the upward region of the droplet. That asymmetry clearly displays a vapor tail dragged by the surrounding hot gas. By disregarding the center of the LIF profile where signal mainly originates from liquid, it is possible to quantitatively retrieve the spatial extent of gaseous aluminum, as presented in Figure 12c, for each side of the droplet. Aluminum signal is divided by 100 and disappears at 3 radii on the lower side, whereas it lasts for 6.5 radii on the upper side. It would be useful to compare such extents to numerical simulations of single-droplet combustion from [7,21,27]. However, it is important to note that simulations are performed in a simplified environment with oxidation at 1 bar. The relevance of our high spatial and temporal resolution LIF imaging is demonstrated here in a propellant combustion environment. 

**Figure 13:**
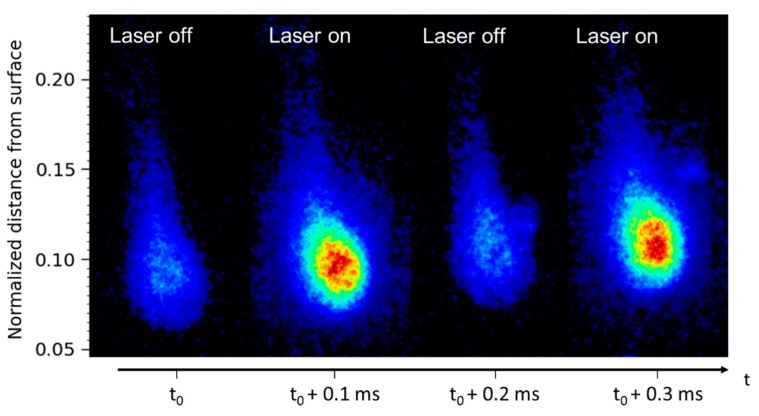
Sequence of four emission and LIF images at h ~1 mm. The distance from surface is normalized to the total flame height. The time delay between successive images is 0.1ms. Images are recorded during the combustion of a burning Al/AP/HTPB propellant sample at 1.0 MPa. The dynamic of the signal is 0–200 counts.

Figure 13 shows expanded views of a droplet located higher in the flame, at h = 1 mm above the propellant surface. The center of the droplet is still visible, in addition to the gaseous extent. In contrast to Figure 12a, the emission of this droplet is stronger (on the same vertical scale) which can be associated with the combustion process, including alumina smoke production which is clearly visible and similar to the behavior of Figure 2b and Figure 4b. Since the droplet is burning at this height in the hot-gas flow-field, the alumina smoke is dragged upward, as previously observed in Figure 2. Moreover, when the laser is on, the aluminum droplet core is clearly visible with good contrast. This adds consistency to the set of observations in our paper, both low on the propellant surface and higher in the flame. 

## 4. Conclusions

The aim of this paper was to apply Al-PLIF to study aluminum combustion in solid-propellant flames. The method yielded interesting information, such as the distribution of atomic aluminum around a molten particle during combustion at high pressure, up to 1.5 MPa. Thanks to careful selection of the excitation/detection scheme in the Al spectrum, the use of atoms in propellant flame as natural tracer was demonstrated to be more pertinent than targeting molecules. The high efficiency of metallic fluorescence is particularly useful in high pressure flames since signal strength remains sufficiently high. Comparison between emission (without laser) and LIF images (with laser) showed a LIF SNR as high as 15. Recording images with a laser wavelength detuned from Al resonance confirmed that there was no contribution of Mie/Rayleigh scattering. Background luminosity was thus mainly dominated by blackbody emission. Due to the high sensitivity of Al fluorescence, both the position and velocity of particles can be retrieved from the images. Velocity measurements presented in this paper are in good agreement with those obtained from the shadowgraphy technique. Shadowgraphy, emission, and LIF diagnostics are thus complementary to understanding the physicochemical processes that a droplet undergoes, and hence provide a good insight into aluminum combustion. A wider field of view enabled us to follow droplets high enough in the flame for the first time. As demonstrated, high sampling rates (10 kHz) are well-suited to following droplet evolution during the combustion run. Information was further improved by the use of a long-distance microscope objective to improve the spatial resolution of the images. Comparisons between emission and LIF images provided in situ observations of the heating/ignition/combustion of Al particles and of gaseous aluminum diffusion from its liquid core. This made it possible for droplet behavior to be analyzed at various heights above the propellant surface for the first time. In the future, due to the high number of images, it will be necessary to use automatic statistical techniques that may help to recognize the flame (in large images) and to better discriminate LIF from emission droplets. Droplet detection and tracking algorithms are currently under development. Improvements in measurement accuracy may lead to credible new data about the very inhomogeneous combustion behavior in the surroundings of any particle.
